# Revisiting a Faculty Career Management Life Cycle Model: Anticipating and Navigating Career Transitions in Academic Medicine

**DOI:** 10.1007/s10880-024-10054-0

**Published:** 2024-11-15

**Authors:** Troy S. Buer, Michele A. Kutzler, Abbie Salcedo, Barbara Overholser, Susan M. Pollart, Nancy D. Spector

**Affiliations:** 1 Office of Faculty Affairs and Faculty Development, University of Virginia School of Medicine, Charlottesville, VA USA; 2 Department of Medicine, Drexel University College of Medicine, Philadelphia, PA USA; 3 Executive Leadership in Academic Medicine®, Drexel University College of Medicine, Philadelphia, PA USA; 4 Department of Family Medicine, Office of Faculty Affairs and Faculty Development, University of Virginia School of Medicine, Charlottesville, VA USA; 5 Department of Pediatrics, Executive Leadership in Academic Medicine®, Drexel University College of Medicine, 2900 W. Queen Lane, K-Wing, Philadelphia, PA 19129 USA

**Keywords:** Faculty, Career, Life cycle, Transitions, Vitality

## Abstract

Career management models are valuable tools for faculty pursuing a career in academic medicine. These models help faculty transition through various stages of their careers, including commonly pursued academic advancements from assistant professor to full professor, as well as less common transitions like moving from full-time to part-time status, taking sabbaticals, going on medical leave, or assuming executive leadership roles. The success of faculty members across these stages is influenced by both environmental factors and individual-level characteristics. Recognizing career stages and transitions, as well as the impact of personal and environmental factors on career growth, is crucial. The proposed Faculty Career Self-Management Model (FCSM) provides a visual and descriptive framework to guide individual faculty and the professionals who support them in understanding, preparing for, and navigating career stages and professional transitions to build and sustain meaningful careers in academic medicine. The FCSM serves as a framework to explore, develop, and share best practices in supporting faculty vitality across the career lifespan.

## Introduction

Career transitions are inevitable for faculty given the accepted (and expected) professional path of assistant, associate, and full professor (Austin, 2010). Faculty in academic medicine are often faced with potentially career-altering decisions as they experience traditional (i.e., academic advancement) and non-traditional (i.e., transition from faculty member to administrator) transitions. Given that the path to success in academic medicine can be tedious and demanding (Richter et al., 2020), understanding how to navigate professional transitions is essential for a rewarding academic career (Viggiano & Strobel, 2009). Career evolutions present personal and professional opportunities and challenges while potentially allowing faculty to reinvent themselves across the faculty life cycle. A lack of knowledge and skill in effectively navigating the transition process may lead to poor career decisions and negative physiological responses (i.e., elevated state of depressive symptoms, heightened anxiety levels, sleep disturbance), thereby impacting job performance and satisfaction. Faculty, therefore, must be prepared to anticipate, respond to, and engage with the inevitable personal and professional challenges and opportunities they will face while pursuing career goals.

Career management models are helpful tools to aid faculty going through career transitions (Smith & Bunton, 2012). These frameworks can serve as practical guideposts to assist faculty members and faculty affairs professionals in anticipating and navigating the decision points associated with career building. After introducing four career management frameworks, we integrate elements from each model to propose a *Faculty Career Self-Management Model* (FCSM). The goal of the FCSM is to help faculty, and faculty affairs professionals who support them, anticipate, and navigate career stages and professional transition periods to build and sustain a career in academic medicine. The FCSM model aims to function as a guide for academic leadership in establishing needed resources to ensure faculty possess the tools for professional success across their career lifespan. The FCSM highlights career stages, personal and environmental factors impacting transitions, and institutional support to faculty. The proposed model centers faculty vitality as the core goal of career management regardless of career stage. Importantly, the FCSM also recognizes opportunities for faculty to exit and re-enter academic medicine and acknowledges personal and environmental factors that can impact career development and equity in transition. These elements render the proposed FCSM model unique compared to the four career management frameworks presented in the current article.

### Career Management Life Cycle Model

The Career Management Life Cycle Model was designed to foster faculty vitality by helping individual faculty recognize and respond to the core career phases they would expect to encounter from recruitment through retirement (Viggiano & Strobel, 2009). Recruitment is focused on aligning individual and institutional needs, goals, motivations, and capabilities. Orientation provides new faculty with successful transition, socialization, and integration experiences. Exploration helps faculty consider professional opportunities and available career support resources. Engagement helps faculty choose a path, set career goals, and create relevant professional development plans. Development includes all the activities, resources, and opportunities to accomplish goals and develop competence. Faculty move to the vitality phase as they achieve the “optimal capability of the individual to make significant and meaningful contributions to their career goals and the institution’s missions” (Viggiano & Strobel, 2009, p. 77). Career growth and transition occur when individuals experience major career changes whether they be planned, emergent, or unplanned. Faculty in transition benefit from organizational support including mentoring, coaching, and development plans. Retirement/emeritus is the planned move away from active and ongoing institutional service (Viggiano & Strobel, 2009).

**Fig. 1:**
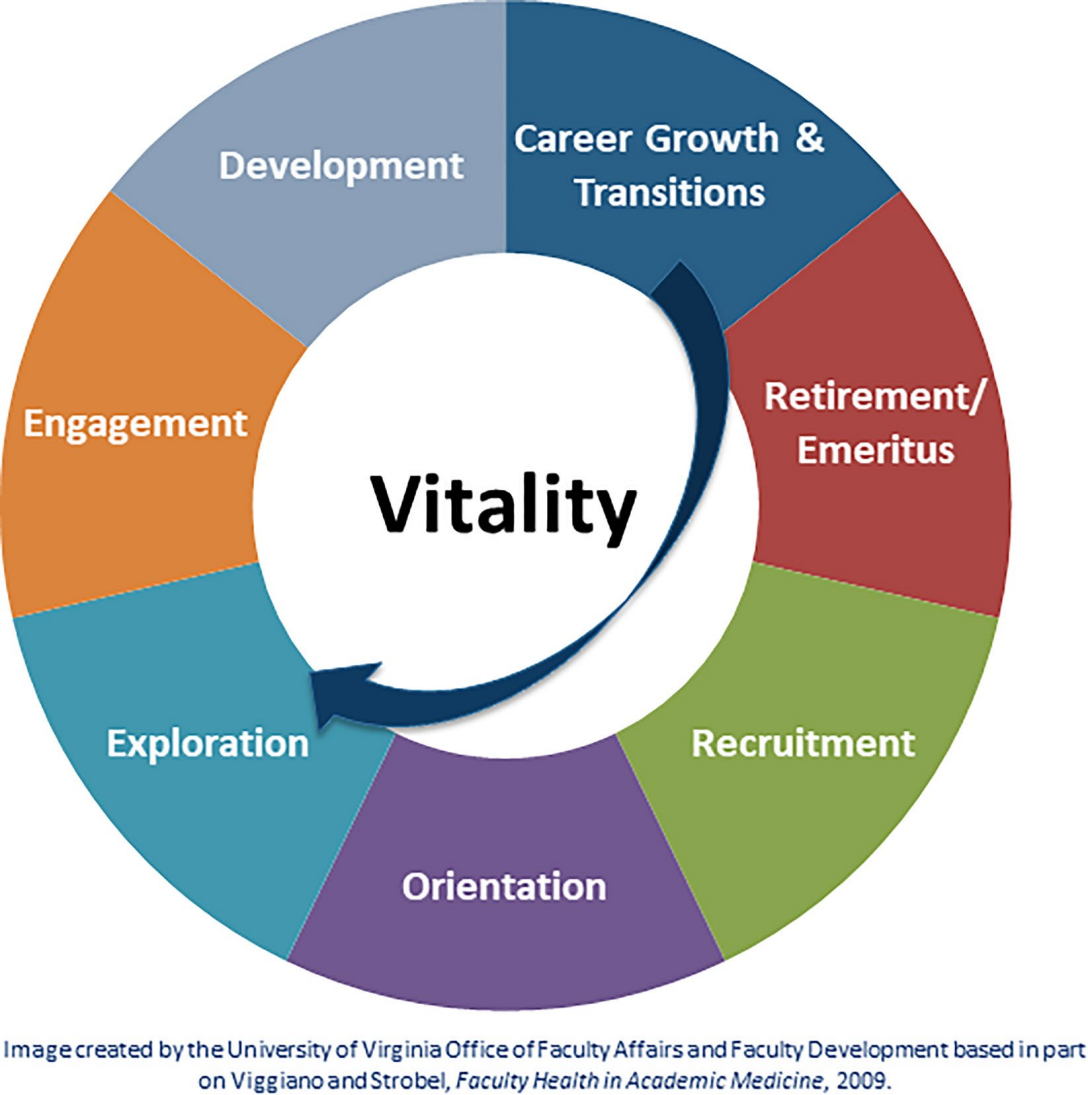
Faculty Career Management Life Cycle Model. Established in 1994, the School of Medicine’s faculty development program is designed to provide professional development and learning opportunities to support faculty throughout their careers as educators, researchers, and physicians. The visual illustrates career phases, places vitality at the center, and adds an arrow to show the windows of opportunity faculty must re-invent themselves through career growth as they encounter and engage with new and unanticipated opportunities. Institutional support of faculty can help ensure vitality across a career lifespan by fostering and encouraging faculty motivation, autonomy, and meaningful work. Career development and engaging in new professional opportunities across a career lifespan are keys to maintaining faculty vitality.

The University of Virginia School of Medicine created a visual based in part on Viggiano and Strobel’s (2009) work (Fig. 1). The visual illustrates career phases, places vitality at the center, and adds an arrow to show the windows of opportunity faculty must re-invent as they engage with new and unanticipated opportunities. Rather than moving directly from career growth/ transitions to retirement, faculty can re-enter and explore new areas, moving to a focus of engagement iteratively multiple times during their career. Institutional support of faculty can help ensure vitality across a career lifespan by fostering faculty motivation, autonomy, and meaningful work. Career development and engaging in new professional opportunities across a career lifespan are keys to maintaining faculty vitality.

### Career Learning Cycles

Hall and Chandler (2008) conceptualized careers as a series of learning cycles. These career cycles are composed of four specific stages. Individuals first consider potential professional opportunities in the exploration stage and move to the trial stage once they choose and begin to engage in a career opportunity or path. During the establishment stage, individuals experience role integration by learning about the role and exhibiting skill development in required capabilities. Individuals ultimately move to the proficiency stage as they are fully engaged in the position and have demonstrated role and interpersonal competence. Proficiency leads to transitions, as individuals consider and explore new professional opportunities, which then begins a new learning cycle. Individuals build upon existing skills across learning cycles and develop new professional competencies. Relationships play an essential role in triggering and facilitating (or hindering) successful movement through a career learning cycle. Mentors, coaches, and sponsors as well as individual personal relationships (family, friends, and colleagues) play critical roles in providing career and psychosocial support during transitions. The amount and quality of support faculty receive, particularly developmental relationships, influences how smoothly they experience moving through career learning cycle stages (e.g., engagement, satisfaction, stress, role adjustment, etc.) (Baker & Terosky, 2017).

### Social Cognitive Model of Career Self-Management

The Social Cognitive Model of Career Self-Management (CSM) moves beyond career phases and stages to explore how individuals make career choices and manage their careers. The CSM framework emphasizes adaptive career behaviors individuals can use to succeed in diverse work environments throughout their careers (Lent & Brown, 2013; Lent et al., 2016). Adaptive career behaviors, such as career exploration, decision-making, retirement planning, and identity management, empower individuals to actively participate in their career development, navigate transitions, and achieve career goals. These actions are connected to the core social cognitive variables of self-efficacy, outcome expectations, and goals. Self-efficacy refers to beliefs about one's ability “to manage specific tasks necessary for career preparation, entry, adjustment, or change across diverse occupational paths” (Lent & Brown, 2013, p. 561). Outcome expectations are the beliefs about positive or negative “anticipated consequences” when deciding to take specific actions (Lent et al., 2016). Goals are the plans to engage in a specific adaptive career behavior such as exploring new leadership roles. Finally, the CSM model also explores how adaptive career actions are hindered or facilitated by the interplay of individual-level traits (e.g., gender, race/ethnicity, personality characteristics, etc.) and environmental supports and barriers (e.g., mentors, resources, reactions of others, etc.) (Lent et al., 2016). The interactive relationship between person, environment, and behavior is known as “triadic reciprocity” as each element influences the others (Fasbender & Deller, 2017).

### Schlossberg Transition Model

Schlossberg’s Transition Model (Anderson et al., 2021; Schlossberg, 2011) provides a framework for understanding how individuals can navigate life and career transitions. The primary step in dealing with change is to clarify which one of the model’s three types of transitions is occurring. Anticipated transitions are predictable and planned (e.g., academic promotion). Unanticipated transitions are unplanned and potentially unpredictable (e.g., layoff). Non-event transitions are expected to occur but ultimately do not happen. For example, faculty may not achieve anticipated promotions or be selected for desired leadership roles. Navigating transitions varies by individual based on the resources or deficits each person brings to their transition event across four domains. Schlossberg refers to these as the Four S’s: situation, self, support, and strategies. Situation refers to the individual’s situation when the transition occurred (e.g., transition trigger, transition timing, personal control of transition, role change, duration, experience, concurrent stress, and assessment of who/what is responsible for transition). Self refers to personal and demographic characteristics that may impact how well the person copes with the situation. These include age and stage of life, gender, race/ethnicity, health, socioeconomic status, and psychological factors such as resilience, optimism, etc. Support includes the availability and strength of personal relationships, developmental networks, and professional associations. Strategies are the approaches used to cope with the transition. For example, the individual could try to alter the situation through negotiation, ask for advice, reframe the change, create a new development plan, or deal with the transition in positive ways through exercising, journaling, etc.

These four frameworks provide insight into career and transition stages as well as the personal and contextual factors that may facilitate or hinder successful career decision-making, advancement, and professional transitions. Table 1 summarizes the key elements of each model integrated into our proposed model.

**Table 1:** Key elements of four career management and transition frameworks

Career management life cycle model (Viggiano & Strobel, 2009)	•Model helps institutions support faculty in distinct career phases to ensure they achieve and sustain vitality. The model describes experiences, challenges, and needs faculty may encounter across core career phases: recruitment, orientation, exploration, engagement, development, vitality, transition, and retirement •Key definition: Vitality is the “optimal capability of the individual to make significant and meaningful contributions to their career goals and the institution’s missions” (p. 77)
Career learning cycles (Hall & Chandler, 2008)	•Careers are conceptualized as a series of four-stage learning cycles: exploration, trial, establishment, and proficiency •Once proficiency is achieved, individuals enter a new learning cycle to develop new skills and build on the competencies already achieved •Career learning cycles can be triggered by individual-level factors (personal goals, motivations, capabilities, etc.); work-level factors (supervisor, new role, developmental networks, etc.); and organization/society-level factors (economy, world events, technology, etc.)
Social cognitive model of career self-management (Lent & Brown, 2013)	•Explores the process for how individuals manage their careers (e.g., developmental tasks, challenges, opportunities, etc.) •Emphasizes adaptive career behaviors as ways individuals are empowered to actively participate in their career development, navigate transitions, and achieve career goals •Explores how individual-level and contextual variables impact self-efficacy, outcome expectations, and goals •Describes how self-efficacy, outcome expectations, and goals shape adaptive actions
Schlossberg transition model (Schlossberg, 2011; Anderson, Goodman, Schlossberg, 2021)	•Provides a framework for understanding how individuals can navigate life and career transitions •Defines three types of transitions: anticipated transitions are predictable and planned; unanticipated transitions are unpredictable and unplanned/not scheduled; non-event transitions were expected to occur but did not •Introduces the Four Ss as factors that influence how well an individual will be able to navigate transitions: ◆Situation: transition triggers, duration, timing, level of control, concurrent stress ◆Self: personal characteristics (e.g., age and life stage, gender, socioeconomic status, race/ethnicity, etc.) ◆Support: social support (family, friend, colleagues, etc.) and psychological resources (e.g., optimism, resilience, self-efficacy, etc.) ◆Strategies: coping response (e.g. reframe, gather information, negotiate, stress management, etc.)

### A New Faculty Career Self-Management Model

**Fig. 2:**
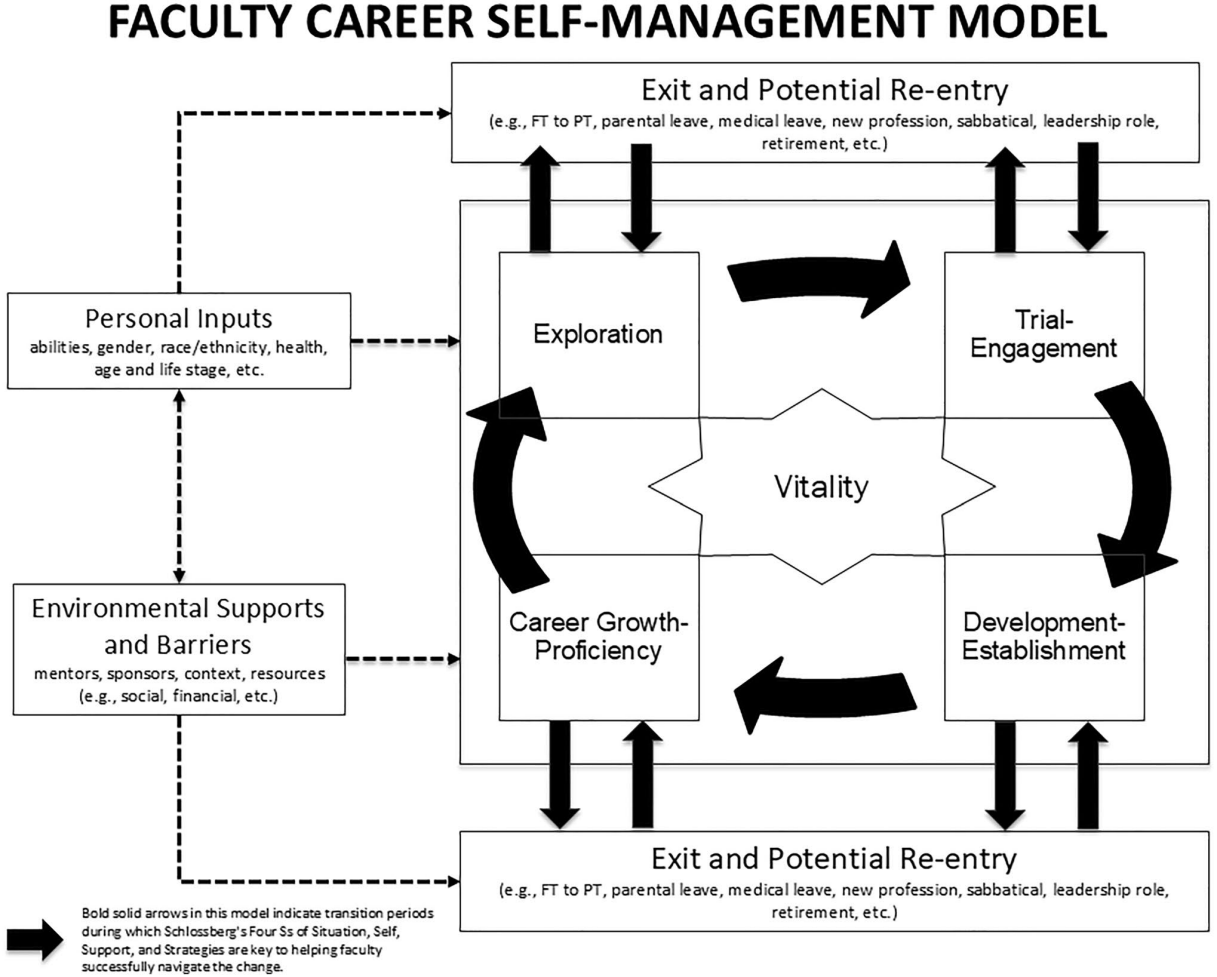
Faculty Career Self-Management Model. The Faculty Career Self-Management Model (FCSM) serves as a framework to explore, develop, and share best practices in supporting faculty across career stages and professional transitions. The FCSM provides a visual and descriptive guidepost for faculty in academic medicine, and the faculty affairs professionals who support them, to focus on and foster faculty vitality across career stages and transition periods. As a function of satisfaction, engagement, and productivity, faculty vitality is at the center of the FCSM to emphasize that faculty can be vital within each career stage and during periods of transition. Faculty affairs professionals and institutions are essential to fostering vitality across the faculty life cycle. Personal inputs, and environmental supports and barriers influence each other and impact how individuals experience each career stage and transition period. Institutional support of faculty can help ensure vitality across a career lifespan by fostering and encouraging faculty motivation, autonomy, and meaningful work. The FCSM includes four core career stages: Exploration, Trial-Engagement, Development-Establishment, and Career Growth-Proficiency. These stages are sequential and continuous thereby fostering professional growth over the lifespan of a faculty member’s career. The FCSM recognizes the autonomy faculty have to reinvent themselves as they move across career stages, engage with new professional opportunities, and overcome professional or personal challenges. Legend: Full Time (FT); Part Time (PT).

Integrating elements from each of the models previously described, we propose a career management framework for faculty in academic medicine: the Faculty Career Self-Management Model (FCSM). The FCSM serves as a framework to explore, develop, and share best practices in supporting faculty across the career lifespan. The FCSM provides a visual and descriptive guidepost for faculty, and the faculty affairs professionals who support them, to focus on and foster faculty vitality across career stages and transition periods (see Fig. 2). Faculty vitality is the “synergy between high levels of satisfaction, productivity, and engagement that enables the faculty member to maximize [their] professional success and achieve goals in concert with institutional goals” (Dankoski et al., 2012, p. 635). As a function of satisfaction, engagement, and productivity, vitality is at the center of the FCSM to emphasize that faculty can be vital within each career stage and during periods of transition. Faculty affairs professionals are essential to fostering vitality.

The FCSM can help faculty prepare for and navigate core career stages and transition opportunities. Combining Viggiano and Strobel (2009) and Hall and Chandler (2008) the model includes four core career stages: Exploration, Trial-Engagement, Development-Establishment, and Career Growth-Proficiency. These stages are sequential and continuous thereby fostering professional growth over the lifespan of a faculty member’s career. The FCSM recognizes the autonomy faculty have to reinvent themselves as they move across career stages, engage with new professional opportunities, and overcome challenges. Importantly, as shown in Fig. 2, the proposed model recognizes opportunities for faculty to exit and re-enter academic medicine (e.g., sabbatical, parental leave, medical leave, full-time (FT) to part-time (PT) or re-entry into the workforce, transition to another industry or leaving the workforce, etc.). Faculty may re-enter at any stage and begin to re-vitalize their careers by accessing transition support from institutions and personal developmental networks and relying on their own personal characteristics like resilience and conscientiousness (Lent & Brown, 2013; Schlossberg, 2011; Viggiano & Strobel, 2009). The success of professional transitions is shaped by the personal and institutional resources faculty bring to planned or unanticipated transitions (i.e. Schlossberg Four S’s).

Following Lent and Brown (2013) and Schlossberg’s Four S’s (Anderson et al., 2021), the FCSM recognizes that vitality and equity in professional growth, career choices, and transitions are impacted by individual characteristics and environmental factors. Individual and institutional factors predict faculty vitality (Dankoski et al., 2012). Faculty have personal characteristics and realities that impact their careers. Faculty live and work within personal and professional contexts that may provide differential access to individuals, groups, or resources that support or hinder career growth. Faculty career transitions, particularly for individuals from minority groups, represent a complex journey as they encounter barriers and challenges. For example, institutions may have a culture that is not inclusive of women and faculty of color. This may manifest in a lack of representation in leadership positions, a lack of mentorship, and a hostile or discriminatory environment (Lin & Kennette, 2022). Individuals may hold unconscious biases that disadvantage women and faculty of color. This bias can manifest in microaggressions, prejudice, and discrimination (Calder et al., 2023). Structural barriers, such as lack of childcare or flexible work arrangements, can make it difficult for those in caregiving roles, most often women, to succeed in academia (Williams, 2021). The competitive environment of academia can create additional challenges for women and faculty of color who may have to work harder to get the same recognition and opportunities as their White male peers (Ferrero, 2020).

While these examples do not exhaust all the obstacles minority groups face, it is crucial to recognize the slow rate of progress despite gains made (Martínez-Blancas et al., 2022). By intentionally and actively addressing (i.e., identifying organizational policy obstacles to address issues of recruitment and retention of minority groups) these and other race, ethnicity and gender-based barriers leaders can cultivate a more creative and innovative academic environment (Mangurian et al., 2024). According to Freeman and Huang (2014), this could lead to contributions from different cultures, creating an improved, more altruistic departmental climate.

The FCSM encourages collaborative institutional efforts to create a more inclusive and equitable environment by including the concept of triadic reciprocity. This element emphasizes how individual behavior, personal inputs, and environmental supports and barriers influence each other and impact how individuals experience each career stage and transition period (Fasbender & Deller, 2017). Leaders can use this concept to recognize the needs of faculty across groups and provide needed support to help them thrive. Equitable criteria for compensation, advancement, and productivity coupled with opportunities for mentoring and sponsorship, professional development, engaging in new professional opportunities, and experiencing professional achievement and recognition foster faculty vitality across the career life cycle (Shah et al., 2018; Viggiano & Strobel, 2009).

Faculty experience many career transitions, including being appointed as a first-time faculty member; earning academic rank promotions; holding various leadership appointments; and, ultimately, transitioning out of employment. In the following section, we highlight how the FCSM can be used to guide two types of transitions: firstly, transitions related to non-linear careers (i.e. part-time or workforce re-entry); secondly, late career transitions in academic medicine.

### Career Transitions Related to Non-linear Careers

Transitions related to non-linear academic careers may include instances of leave (medical, parental, sabbatical), transitions to non-academic careers, and also may include faculty who move to part-time. Part-time faculty members are valuable institutional resources; however, they require flexible work schedules. Institutions should identify models to leverage their skills and interests. Pollart et al. (2015) recommend that we as faculty affairs leaders customize individuals’ career paths to “recognize the needs and desires of today’s workforce, which includes more women, who typically have nonlinear careers; more men seeking a change in work demands particularly late in their career; and changing family structures, which must accommodate the needs of two adults in the professional workforce” (p. 358). Benko and Weisberg (2007) introduce the idea of a "career lattice" instead of a "career ladder." This concept supports both horizontal and vertical movement within an organization's hierarchy throughout a person's career. This model is amplified by Pollart et al. (2015) since it allows for career-spanning adjustments in workload and schedule with corresponding adjustments in organizational role and pace of professional advancement. Institutions will benefit if they can recruit and retain these faculty members who desire a nontraditional professional path, support and development throughout their careers, and make allowances for graceful movements within a lattice of professional obligations and opportunities (Pollart et al., 2015).

### Late Career Transition

Academic medicine has undergone substantial transformations over the years. As the field embraces advancements in technology, shifts in healthcare delivery, and changes in educational paradigms, faculty members find themselves at the intersection of tradition and innovation. Late-career transitions in this dynamic environment require a nuanced understanding of how to navigate changing expectations and contribute meaningfully to the academic community. However, late-career transitions are not without challenges. Faculty members approaching the latter stages of their professional lives grapple with issues related to identity shifts, succession planning, mentorship dynamics, and institutional expectations. These problems highlight the importance of doing a thorough analysis of the factors that affect late-career transitions and the creation of supporting initiatives within academic institutions. One of the primary challenges faced by faculty in late-career transitions is the shift in professional identity. Accustomed to well-established roles as educators, researchers, or clinicians, faculty members must navigate the process of redefining their identities beyond their traditional academic roles. In a qualitative study conducted by Onyura et al. (2015), 21 faculty across eight specialties (clinical and surgical) were interviewed about late-career and retirement planning issues and “four primary themes were identified: the centrality of occupational identity, experiences of identity threat, experiences of aging in an indifferent system, and coping with late-career transitions” (p. 794). Furthermore, the findings of the study indicated that apprehensions regarding identity risks materialized in matters about emotional detachment, self-esteem following retirement, the maintenance of professional skills and clinical expertise, and a perceived lack of purpose leading to feelings of alienation. According to the study, the decisions about retirement were notably impacted by these identity challenges (Onyura et al., 2015). Organizational and systemic support were found to be inadequate. The article highlighted how their participants utilized a variety of coping mechanisms, including reorienting their priorities, integrating new activities, and envisioning and reassessing different facets of themselves. This coping mechanism allows late-career faculty to explore new roles within academia. For example, faculty members may engage in advisory roles, interim leadership roles, participate in interdisciplinary collaborations, or contribute to community outreach programs. With vitality at its core, our model recognizes the need for tailored support mechanisms, including mentorship programs, succession planning, and flexible retirement options. By addressing these challenges thoughtfully, late-career transitions can become a phase of renewal, knowledge transfer, and legacy building, enriching academic medicine.

### Individual-Level Opportunities to Ensure Transition or Re-entry Success

Faculty can prepare and gain skills to help navigate career transitions in several ways so that they can enjoy productive and satisfying careers (Table 2). It is important for faculty to establish good behaviors for academic success which are further discussed in the following references (AAMC/GWIMS, 2023; Skarupski, 2020) and include: (a) assembling and revisiting one’s mentoring mosaic of junior, peer and senior faculty mentors (Cruz et al., 2015); (b) developing operational, personal and professional networks (building a sphere of influence, scholarly focus, and research collaboration); (c) building an effective research team; (d) actively engaging with professional societies; (e) learning and implementing foundational teaching skills and principles; (f) creating and committing to professional and leadership development planning (Spector & Sectish, 2012); (g) learning how to balance institutional “citizenship” and saying yes as well as no when aligned with mission, vision and values; (h) learning to navigate the departmental and institutional sociopolitical landscape; (i) connecting with sponsors; (j) gaining negotiation skills and tools for navigating difficult conversations.

**Table 2:** Opportunities to enhance vitality

Personal inputs	Environmental supports
Assembling and revisiting one’s mentoring mosaic of junior, peer, and senior faculty mentors	Organizing new faculty orientation and effective onboarding programs
Networking to develop operational, personal, and professional networks (building sphere of influence, scholarly focus, and research collaboration)	Creating faculty, professional, and leadership development programs and ensuring dedicated resources and leadership buy-in
Building effective research teams	Expanding available faculty tracks to align with diverse types of faculty
Engaging in professional societies	Aligning and ensuring transparency of promotion criteria with an institutional mission
Participating in faculty development programs as an educator	Implementing work schedule flexibility to retain promising part-time faculty as a pipeline to high-quality workforce
Conducting professional development planning	Conducting needs assessments of mid-and senior level faculty members and develop programming to meet needs
Seeking continual leadership development training at all levels of faculty rank	Creating institutional coaching programs
Aligning work with one’s mission, vision, and values	Re-investing and re-imagining late-stage career opportunities for senior faculty
Navigating sociopolitical landscape	Planning for succession and leadership pipeline for senior faculty and senior administrators
Utilizing sponsorship networks	Identifying organizational policy obstacles to address issues of recruitment and retention of minority groups
Gaining negotiation skills and tools for navigating difficult conversations	Creating and implementing policies to support childcare, eldercare, and other caregiving responsibilities
	Conducting exit and stay interviews with faculty

### Institutional Systems and Policies Critical for Faculty During Transitions and Re-entry Into Academic Medicine

Identifying organizational policies that support the infrastructure needed and use of evidence-based interventions and professional development programming to support intentional career development of faculty is critical for the recruitment and retention of outstanding faculty and ensuring faculty vitality. Institutional support of faculty can help ensure vitality across a career lifespan by fostering and encouraging faculty motivation, competence, autonomy, and meaningful work (Dankoski et al., 2012; Pink, 2011; Viggiano & Strobel, 2009). Regardless of one’s profession, career transitions are inevitable. A study conducted by the Association of American Medical Colleges (AAMC) revealed that while leadership may believe that faculty are thriving and have the infrastructure, they need to be successful, faculty feel siloed and lost navigating institutional processes (Dandar et al., 2017; Pololi et al., 2015). In addition, they may feel unable to adequately perform their jobs in the absence of institutional systems and policies to support them. Challenges with navigating institutional processes further confound productivity, and create misalignment of personal values with institutional missions, general job dissatisfaction, and professional burnout, all of which may lead to job turnover (Pololi et al., 2015; Shanafelt & Noseworthy, 2017). Individuals with job satisfaction, high organizational engagement and commitment, and continual organizational support have reported lower intentions to leave their environment (Holtom & Inderrieden, 2006).

Institutions and offices of faculty affairs and development can support faculty during career transitions and re-entry into academic medicine in several ways (Table 2) to provide environmental support. Examples of organizational policies, practices, and programming to ensure faculty recruitment, vitality, and retention include (a) offering orientations and onboarding activities ensuring a diverse network for community building; (b) including faculty, professional, and leadership development programs (Kutzler et al., 2021); (c) ensuring dedicated resources and leadership buy-in; (d) expanding on available faculty tracks to align with diverse types of faculty (employed, volunteer, part-time, community clinicians, faculty transitioning from non-academic careers back to academia, tenure track basic science); (e) aligning and ensuring transparency of promotion criteria with an institutional mission; (f) implementing work schedule flexibility to retain promising part-time faculty as a pipeline to high-quality workforce (Pollart et al., 2015); (g) conducting needs assessments of mid-and senior level faculty members and develop programming to meet needs; (h) creating institutional coaching programs; (i) re-investing and re-imagining late-stage career opportunities for senior faculty; (j) succession planning for senior faculty and senior administrators; (k) identifying organizational policy obstacles to address issues of recruitment and retention of minority groups, as well as creating and implementing policies to support childcare, eldercare, and other caregiving responsibilities (Sosa & Mangurian, 2023) and exit and stay interviews with faculty.

### Implications for Psychologists in Academic Health Centers

The FCSM is intended to help faculty build and sustain vital careers by guiding how they understand, prepare for, and navigate career stages and professional transitions. This model can benefit faculty across academic medicine disciplines, including psychologists. Psychologists have an ever-increasing presence across academic medicine being employed across a diverse set of departments including Psychiatry/Behavioral Sciences, Pediatrics, Neurology, Family/Health/Community, and other medical specialties including Rehabilitation/pain management and other medical specialties (Robiner et al., 2014; Sanders et al., 2010). For psychologists working in academia across clinical, research, and education missions, academic promotion requires a strategic plan to help navigate the challenges and rewards, early, mid-career, and late-career transitions, and professional exits and re-entries. As noted by Sanders et al. (2010), advice specific to each stage of career development (early, mid, and late) includes themes of coaching and teamwork that are critical to success in academia. Thus, the FCSM can guide individual faculty to anticipate stage-specific support needs and academic leadership to establish the necessary resources to ensure that faculty members possess the appropriate tools and support systems for optimal success.

The FCSM is also of interest to psychologists as leaders in academic medicine given the expanded administrative and service roles, they have assumed that directly impact faculty vitality. Shaffer et al., 2021 and Smith & Bunton, 2012 have noted that psychologists are well-prepared to advocate for faculty vitality as institutional leaders of faculty affairs offices and as members of key AAMC committees and organizations such as the Council of Faculty and Academic Societies, Group on Faculty Affairs, and Group on Diversity and Inclusion. In these roles, psychologists, along with faculty from other disciplines in similar leadership positions, also provide mentorship, coaching, and sponsorship, create professional development initiatives, and seek to address workplace stressors, mitigate burnout, and enhance faculty well-being and vitality (Hill et al., 2022; Shaffer et al., 2021; Sim et al., 2023; Williams et al., 2020). The AAMC is a “valued resource for developing ongoing awareness of the changing landscape of academic medicine to facilitate continual adaptation for career-rewarding faculty vitality” (Smith & Bunton, 2012, p. 25). In 2023, the AAMC released the “StandPoint™ Surveys: 2023 State of Medical School Faculty Engagement”. Using data collected from approximately 18,000 full- and part-time faculty between late January 2020 and December 2022, this publication highlights insights into the most salient issues impacting faculty engagement in academic medicine and provides recommendations to help medical schools increase engagement and retention of faculty.

## Conclusion

Career management models serve as valuable tools to help faculty navigate career transitions within academic medicine. Due to the changing landscape of AHCs and evidence of strategic and unexpected faculty career transitions, it is imperative that we consider a new faculty lifecycle model and include support systems for exit and re-entries for faculty as described here. As reported in the AAMC StandPoint™ Surveys (Dandar et al., 2023), twelve percent of faculty reported they are likely to retire in the next one to two years, and an additional 26% of faculty reported being somewhat likely, likely, or highly likely to leave their medical school in the next one to two years. Of the 26% of faculty who reported being somewhat likely, likely, or highly likely to leave their medical school in the next one to two years, 47% reported being somewhat likely, likely, or highly likely to leave academic medicine altogether over the next one to two years. Reasons for considering leaving varied by faculty group, and, overall, the three most commonly cited reasons were compensation and benefits, work-life balance and burnout, and professional and advancement opportunities. In addition, a regression analysis showed that the factors driving faculty retention the most were satisfaction with one’s job and the nature of day-to-day work, perceptions around the medical school’s ability to recruit and retain high-quality faculty, one’s opportunities for career growth and professional advancement, the workplace culture, and one’s relationship with their supervisor. The authors recommend future tracking of the types, prevalence, and successful landings for faculty after exit and or re-entry into academic medicine following a transition (exits, re-entries, specific transition data) so that data-driven approaches and best practices can be reported and used to develop innovative programming to better support faculty during transitions. Faculty affairs leaders can use the FCSM to strategize how best to support psychologist faculty members’ careers on a macro level, and psychologists, as human behavior experts, are poised to contribute to institutional leadership (Smith & Bunton, 2012). The institutional climate and culture, faculty collaboration and opportunities for feedback from coaches and mentors, promotion, recruitment and retention, career exit and re-entry into academia, institutional governance and operations, and clinical practice all contribute to faculty satisfaction. Thus, faculty in the field of psychology are well positioned to serve in administrative leadership roles to implement FCSM as a framework to explore, develop, and share best practices in supporting faculty to achieve faculty vitality across the career lifespan.

## Data Availability

Not applicable.
